# Oncolytic Activity of a Chimeric Influenza A Virus Carrying a Human CTLA4 Antibody in Hepatocellular Carcinoma

**DOI:** 10.3389/fonc.2022.875525

**Published:** 2022-04-12

**Authors:** Hao Yang, Guanglin Lei, Fang Sun, Jinxia Cheng, Jin Yan, Shaogeng Zhang, Penghui Yang

**Affiliations:** ^1^ National Clinical Research Center for Infectious Diseases, Fifth Medical Center of Chinese PLA General Hospital, Beijing, China; ^2^ The Graduate Department, Hebei North University, Zhangjiakou, China

**Keywords:** generation hemagglutination, rFlu-huCTLA4, HCC, oncolytic virus, CTLA4 antibody

## Abstract

Oncolytic virotherapy belongs to a kind of active immunotherapy, which could trigger a potent antitumor immune response, showing great potential in clinical application. OVs could induce immune responses through the dual mechanisms of selective tumor killing without destroying normal tissues and induction of systemic antitumor immunity. In this study, we successfully rescued a chimeric oncolytic influenza virus carrying a human CTLA4 antibody in the background of the A/PR/8/34 (PR8) virus. The chimeric virus, called rFlu-huCTLA4, contained the heavy and light chains of the human CTLA4 antibody in the PB1 and PA segments of the PR8 virus, respectively. The first-generation hemagglutination (HA) titers of the rFlu-huCTLA4 virus ranged from 2^7^ to 2^8^, which could be passaged stably in specific pathogen-free (SPF) chicken embryos from P1 to P5. The morphology and size distribution of the chimeric virus were consistent with those of the *wt* influenza virus. The rFlu-huCTLA4 virus could effectively replicate in various cells in time- and dose-dependent manners. ELISA assay revealed that the secreted huCTLA4 antibody levels in chicken embryos increased gradually over time. Furthermore, MTS and crystal violet analysis showed that the selective cytotoxicity of the virus was higher in hepatocellular carcinoma cells (HepG2 and Huh7) than in normal liver cells (MIHA). *In vivo* experiments displayed that intratumoral injection with rFlu-huCTLA4 reduced tumor growth and increased the survival of mice compared with the PR8 group. More importantly, in the rFlu-huCTLA4 group, we found that CD4+ and CD8 +T cells were significantly increased in tumor-bearing BALB/c mice. Taken together, these findings demonstrated that the chimeric oncolytic virus rFlu-huCTLA4 could selectively destroy hepatocellular carcinoma cells *in vitro* and *in vivo* and may provide a promising clinical strategy for targeted immunotherapy of HCC with the oncolytic flu virus.

## Introduction

Primary liver cancer is the second leading cause of cancer-related death, of which hepatocellular carcinoma (HCC) accounts for about 90% of primary liver cancer and is a global health problem (1, 2). Until now, the clinical treatment of liver cancer mainly includes radiofrequency ablation, liver transplantation, hepatectomy, transcatheter arterial chemoembolization (TACE), and systemic therapy. However, one of the main therapeutic strategies for advanced HCC is still molecular targeted drugs represented by sorafenib (3). However, the curative effect of liver cancer is not satisfactory, and the 5-year survival rate was very low (4–6). Therefore, many researchers focus on the research of liver cancer drugs to provide many alternative strategies for the clinical treatment of liver cancer (7–9). Novel treatment strategies to improve the treatment efficacy and the survival rate of HCC are urgently needed.

As one of the new immuno-oncology therapies with the most potential (10), oncolytic virus (OV) is a genetically engineered or naturally occurring virus that could selectively kill tumor cells by activating the immune system without damaging the normal tissues (11, 12). It has been shown that OVs could selectively infect tumor cells and multiply in tumor cells, which cause cell fragmentation and death, then release and spread to infect nearby tumor cells. OVs induced immunogenicity of the target cells and produce an antitumor immune response *in vivo* (13). Currently, investigations on several OVs are encouraging, including vaccinia, coxsackie, adenovirus, reovirus, human herpesvirus 1, and measles, which have been extensively explored and tested in clinical trials for various advanced cancers (14–16). On November 1, 2021, Daiichi Sankyo Company Limited announced that Delytact (Teserpaturev/G47Δ), the third-generation HSR-1 oncolytic virus product, is the first approved oncolytic virus product for the treatment of glioblastoma. It is also the fourth oncolytic virus product approved for listing in the world at present (17). The drug made the world realize the great potential of OVs and greatly promoted the development of oncolytic virus therapy.

Cytotoxic T lymphocyte-associated antigen 4 (CTLA4) is one of the important immune checkpoints, which is a transmembrane receptor on T cells. TCR/MHC (Signal 1) and CD28B7 (Signal 2) co-stimulated T cells to activate. Antigen-presenting cell B7 (including B7-1 and B7-2) bonded the CTLA4 targets on T cells to inhibit T-cell function. Anti-CTLA4 antibodies could block the binding of CTLA4 and B7 to activate T cells (18–21). Previous studies showed that *in vivo* administration of CTLA4 antibodies enhanced antitumor immunity, which has promoted CTLA4 antibodies as immunotherapy for cancer (22–24). As a representative sample of the anti-CTLA4 antibody, ipilimumab has been approved for melanoma therapy by the FDA in 2010, which is an immunomodulatory monoclonal IgG1 antibody directed against the cell surface antigen CTLA4 (25). In addition, tremelimumab is an IgG2 monoclonal antibody that inhibits CTLA4, which has been investigated in several clinical trials and has potential as the next generation of anti-CTLA4 immunotherapy agents (26–28).

In recent years, there have been many studies on targeted and immunotherapy of liver cancer, but few of them have shown unique advantages. Therefore, we innovatively carried out the study on the chimeric huCTLA4 oncolytic virus. In line with this study, we previously reported the generation of a recombinant influenza virus rFlu-CTLA4 encoding mouse the CTLA4 antibody. The rFlu-CTLA4 virus exhibits selective cytotoxicity *in vitro* and inhibits tumor growth in a HepG2 homograft mouse model (29). Due to the low sequence homology of human and mouse CTLA4 antibodies, a chimeric influenza virus encoding the human CTLA4 antibody was generated using reverse genetics. Here, we utilized PB1 viral segments to express the heavy chain and PA viral segments to express the light chain of the human CTLA4 antibody, respectively. Subsequently, the oncolytic efficacy of rFlu-CTLA4 for HCC was investigated *in vitro* and *in vivo*.

## Materials and Methods

### Cells and Viruses

Human hepatocellular carcinoma cell lines HepG2 and HuH7 were obtained from the American Type Culture Collection (Manassas, VA, USA). The normal liver cell line MIHA was obtained from Cell Bank, Shanghai Institutes for Biological Sciences, Chinese Academy of Science (Shanghai, China). COS I cells and MDCK cells were purchased from the Chinese Academy of Sciences; these cells were cultured in Dulbecco’s modified Eagle’s medium (DMEM) (Gibco, Grand Island, NY, USA) containing 10% fetal bovine serum. The use of the cell lines was approved by the Ethics Committee of the Fifth Medical Center of Chinese PLA General Hospital. Wild-type influenza virus A/PR/8/34(PR8) was grown in 9–11 day-old specific pathogen-free (SPF) chicken embryos (Beijing Laboratory Animal Center, China).

### Rescue of Chimeric Oncolytic Virus Expressing the Human CTLA4 Antibody

The sequences of heavy chain and light chain of the huCTLA4 antibody were obtained from GenBank (National Center for Biotechnology Information, NCBI). After optimization, the gene sequences of the huCTLA4 antibody were synthesized by Shanghai Shenggong Biological Engineering Co., Ltd. The recombinant plasmids pFlu-huCTLA4-Basicity polymerase 1(PB1) and pFlu-huCTLA4-Acidity polymerase(PA) were cloned into the pHW2000 vector. Rescue of the chimeric oncolytic virus was performed according to the Effectene transfection kit’s instructions (Qiagen, Hilden, Germany). Briefly, pFlu-huCTLA4-PB1, pFlu-huCTLA4-PA, and the other six plasmids of the PR8 backbone, pHW191-Basicity polymerase 2(PB2), pHW194-Hemagglutinin(HA), pHW195-Nucleoprotein(NP), pHW196-Neuraminidase(NA), pHW197-Matrix protein(M), and pHW198-Non-structural protein(NS), were diluted to 200 ng/μl, then COS I and MDCK cells were co-transfected. After 72h, the allantoic fluid of the chimeric rFlu-huCTLA4 virus was harvested, and the virus titer was determined in HepG2 cells.

### Real-Time PCR Identification

The chimeric oncolytic virus rFlu-huCTLA4 RNA was extracted from the allantoic fluid. Then, RNA was reverse transcribed into cDNA and amplified by PB1 and PA universal primers of the influenza A virus. The primers of PB1 are given below:

Bm-PB1-1:5′TATTCGTCTCAGGGAGCGAAAGCAGGC3′;
Bm-PB1-2: 5′ATATCGTCTCGTATTAGTAGAAACAAGGCATTT3′.

The primers of PA were Bm-PA-1: 5′TATTCGTCTCAGGGAGCGAAAGCAGGTACT3′; Bm-PA-2: 5′ATATCGTCTCGTATTAGTAGAAACAAGGTACTT3′. The PCR products were sequenced and compared with pHW192-PB1, pHW193-PA, pHW197-M, and pHW198-NS of the PR8 virus for agarose gel electrophoresis.

### Electron Microscope

The morphological characteristics and size distribution of rFlu-huCTLA4 were investigated with the electron microscope. The chimeric virus was amplified from the chicken embryo and purified by 30%~60% sucrose gradient centrifugation. Then, the viral morphology and size were observed using the transmission electron microscope after negative staining.

### Virus Growth Curve

The replication ability of rFlu-huCTLA4 was examined on HepG2 and MDCK cells at different time points. The supernatants of 12-, 24-, 48-, 72-, and 96-h cells were collected, and the virus titers were measured at the indicated time point.

### Antibody Purification and Enzyme-Linked Immunosorbent Assay

The 9–11-day-old SPF chicken embryos were infected with rFlu-huCTLA4 and incubated at 37°C for 72 h to harvest allantoic fluid. The antibody is concentrated on the protein G column. The secreted human CTLA4 antibody levels in eggs at the indicated time point were detected with an ELISA Kit (Jianglai Biotec, Shanghai).

### Cell Viability Test

MIHA, HepG2, and Huh7 were respectively spread into 96-well cell boards with a cell number of 1 × 10^4^ cells per hole. The cells were inoculated with rFlu-huCTLA4 at MOI (0.1, 1, 2, 3). After 48, 72, and 96 h. MTS reagent was added to determine the OD value at 490 nm by an enzyme-linked detector.

MIHA, HepG2, and Huh7 cells were planted in 24-well plates. Then the cells were covered with monolayers inoculated with rFlu-huCTLA4 at MOI (0.1, 1, 2, 3). The cells were infected with rFlu-huCTLA4 at MOI values of 0.1, 1, 2, and 3, and negative control was set up. After 48, 72, and 96 h, 1% crystal violet dye was added after the supernatant was discarded, then the results were recorded and analyzed.

Next, we examined the effect of rFlu-huCTLA4 on MIHA and HepG2 cell apoptosis with flow cytometry. The MIHA and HepG2 cells were infected with rFlu-huCTLA4 at MOI of 0.1 and 1. After 48 h, the cells were harvested according to the instructions of the apoptosis kit and detected within 30 min with flow cytometry (BD FACSCalibur, Franklin Lakes, NJ, USA).

### Animal Experiments

All animal experiments were performed under the guidelines of the Institutional Animal Care and Use Committee and Ethics Committee of the Fifth Medical Center of the Chinese PLA General Hospital. All facilities were accredited by the Fifth Medical Center of the Chinese PLA General Hospital.

Mouse hepatoma H22 cells were inoculated into the groin of BALB/c mice to establish a subcutaneous tumor-bearing mouse model. BALB/c mice bearing tumor were divided into 3 groups (8 in each group). Mice were treated when the tumor volume reached about 100–150 mm^3^. Mice were intratumorally injected with PBS, PR8, and rFlu-huCTLA4 at 3 × 10^6^ TCID_50_/100 µl every other day for 7 times.

The PDX mouse model of hepatocellular carcinoma was implanted into the right back of NPI mice with the size of 3 × 3 × 3 mm. When the tumor grew to 80~150 mm^3^, every 8 mice were divided into a group and given 3 × 10^6^ TCID_50_/100 μl for 7 days. The vital signs, survival condition, and tumor volume of mice were observed. When the mice were killed on the 40th day, the tumor tissue was separated and weighed, and observed on pathological sections of the tumor, liver, and lung by HE staining.

### T Lymphocyte Activation

To explore the effect of rFlu-huCTLA4 on the immune system of tumor-bearing mice, spleens of mice were taken on the 7th day after the last inoculation and put into PBS (pH 7.4). The spleen was ground into single cells in PBS containing 2% FBS. Filtration was carried out through a 200-mesh filter, centrifuged at 2,000 r/min for 10 min, and resuspended with PBS. Then CD3^+^, CD4^+^, CD8^+^, CD45^+^, and CD69^+^ antibodies 1 µl each were added to separate the cytotoxic T lymphocytes (CD8^+^CD69^+^) and helper T cells (CD4^+^CD69^+^). Then they were put under 4°C for 30 min then detected by flow cytometry.

### Pathology

The immunohealthy mice were sacrificed after 7 days post virus administration of the last time; the tumors were isolated and fixed with 4% (ml/ml) formaldehyde solution, repaired, dehydrated, dipped in wax, embedded, sectioned, and stained with HE. The growth of tumor cells was observed under the microscope.

### Statistical Analysis

All data were analyzed by GraphPad Prism 8.0 (GraphPad Software Inc., La Jolla, CA, USA). Statistical analyses comparing two groups were performed using Student’s t-test. Three or more groups were compared using analysis of variance, and p < 0.05 was considered significant.

## Results

### Design and Characterization of Recombinant Virus rFlu-huCTLA4

**Figure 1:**
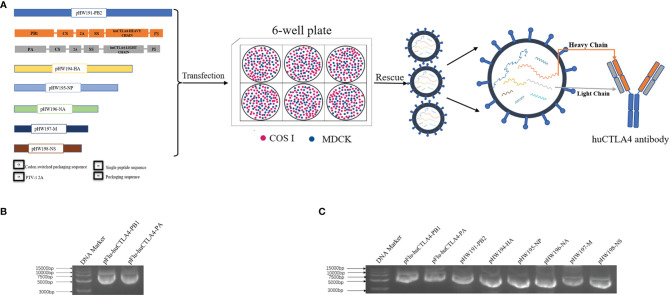
Construction and identification of the recombinant plasmid. **(A)** Schematic diagram of the experimental model: plasmid construction strategy of recombinant oncolytic influenza viruses pFlu-huCTLA4-PB1 and pFlu-huCTLA4-PA. The recombinant plasmid was co-transfected with the remaining 6 skeleton plasmids into MDCK/COSI cocultured cells, and the recombinant oncolytic virus rFlu-huCTLA4 was saved and the huCTLA4 antibody was produced with the replication of the recombinant virus. **(B)** Identification of recombinant plasmids pFlu-huCTLA4-PB1 and pFlu-huCTLA4-PA by agarose gel electrophoresis. **(C)** Identification of 8 transfected plasmids by agarose gel electrophoresis.

The heavy and light chains encoding the huCTLA4 antibody were constructed on PB1 and PA fragments of the PR8 virus, respectively. As illustrated in **Figure 1A**, the recombinant PB1 and PA were cloned into the pHW2000 vector, then co-transfected with the remaining 6 skeleton plasmids to MDCK/COSI-cocultured cells. The recombinant plasmids pFlu-huCTLA4-PB1 and pFlu-huCTLA4-PA were successfully constructed with the sizes of 5,982 and 5,853 bp (**Figure 1B**). The sequence of the recombinant plasmid was consistent with the expected sequence, which indicated that the recombinant plasmid was successfully constructed. The recombinant plasmids pFlu-huCTLA4-PB1 and pFlu-huCTLA4-PA and the other six backbone plasmids pHW191-PB2, pHW194-HA, pHW195-NP, pHW196-NA, pHW197-M, and pHW198-NS of PR8 were transfected into cocultured cells of COS I and MDCK by RG, and the transfection was identified by 8 plasmids (**Figure 1C**).

### Identification of Recombinant Oncolytic Virus rFlu-huCTLA4

**Figure 2:**
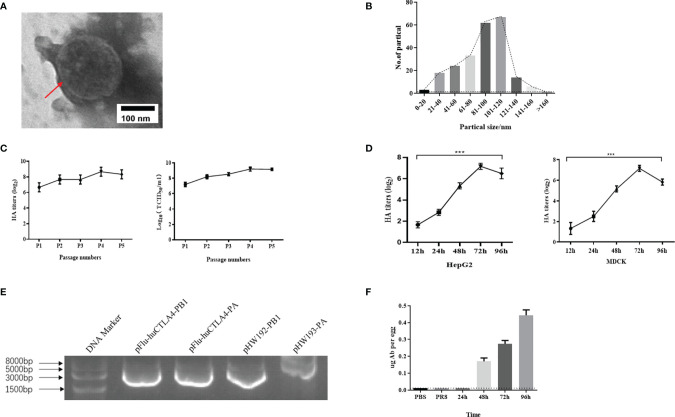
Identification of recombinant oncolytic virus rFlu-huCTLA4. **(A)** The red arrow indicates the virus with regular shape and size, consistent with the morphology and structure of the influenza virus. **(B)** The size of the rFlu-huCTLA4 virus is mainly between 80 and 120 nm. **(C)** rFlu-huCTLA4 was continuously amplified in eggs for 5 generations. The titer of hemagglutination was 2^8^~2^9^ and the titer of the virus was 10^8~9^ TCID_50_/ml after five generations on a chicken embryo. It could be passaged stably in the chicken embryo. **(D)** Growth curve of the recombinant virus. The HA titer of the recombinant virus increased to 2^7^ from 12 to 72 h and decreased to 2^6^ at 96 h. **(E)** Identification of the rFlu-huCTLA4 gene fragment by RT-PCR. **(F)** The antibody content of huCTLA4 detected by ELISA increased gradually with time (***P<0.001, note: the dotted line represents the lowest detection line).

To determine the morphology of the chimeric virus, we observed with the electron microscope that the majority of rFlu-huCTLA4 was spherical. The virus envelope was wrapped on the surface of the nucleocapsid, and there were cilia on the surface of the envelope (**Figure 2A**). The shape and structure of the rFlu-huCTLA4 were consistent with those of the wild-type influenza virus. In addition, the size distribution of the chimeric virus particles was 80~120 nm (**Figure 2B**).

Using reverse genetics, we successfully generated the recombinant oncolytic virus rFlu-huCTLA4. The hemagglutination titer of the first-generation recombinant oncolytic virus, rFlu-huCTLA4, was 2^7^~2^8^. After five generations on a chicken embryo, the titer of hemagglutination was 2^8^~2^9^ and TCID_50_ of the virus was 8~9LogTCID_50_/ml (**Figure 2C**).

rFlu-huCTLA4 was diluted 10^-1^ times to infect HepG2 and MDCK cells. The virus titers were measured in supernatant collected from infected cells at 12, 24, 48, 72, and 96 h. The results showed that the HA titer of the recombinant virus increased to 2^7^ from 12 to 72 h and decreased to 2^6^ at 96 h (**Figure 2D**). Meanwhile, we found that the virus titer had a similar trend, reaching a peak of 10^9^ TCID_50_/ml at 72 h.

The RNA of the rFlu-huCTLA4 virus was extracted from the allantoic fluid by the TRIzol method. After reverse transcription into cDNA, PB1 and PA fragments were amplified by PCR and compared with pHW192-PB1 and pHW193-PA of the PR8 virus. The amplified PB1 and PA fragments of rFlu-huCTLA4 were 2,964 and 2,771 bp, respectively, and the amplified fragments of pHW192-PB1 and pHW193-PA were 2,565 and 2,466 bp, respectively (**Figure 2E**). The PB1 and PA fragments of rFlu-huCTLA4 were consistent with the expected size, indicating that the recombinant oncolytic virus was successfully rescued.

The concentration of the purified antibody was 4.9 ng/ml detected by NanoDrop, and the antibody content was detected with the huCTLA4 antibody ELISA kit. The results showed that no antibody was detected in PBS and PR8 groups, but elevated huCTLA4 antibody levels were detected over time in the rFlu-huCTLA4 group. The huCTLA4 antibody levels of each embryo were 0.17 ± 0.02, 0.27 ± 0.02, and 0.45 ± 0.03 µg at 48, 72, and 96 h, respectively (**Figure 2F**).

### The Selective Toxicity of Recombinant Virus to Various Hepatoma Cell Lines

**Figure 3:**
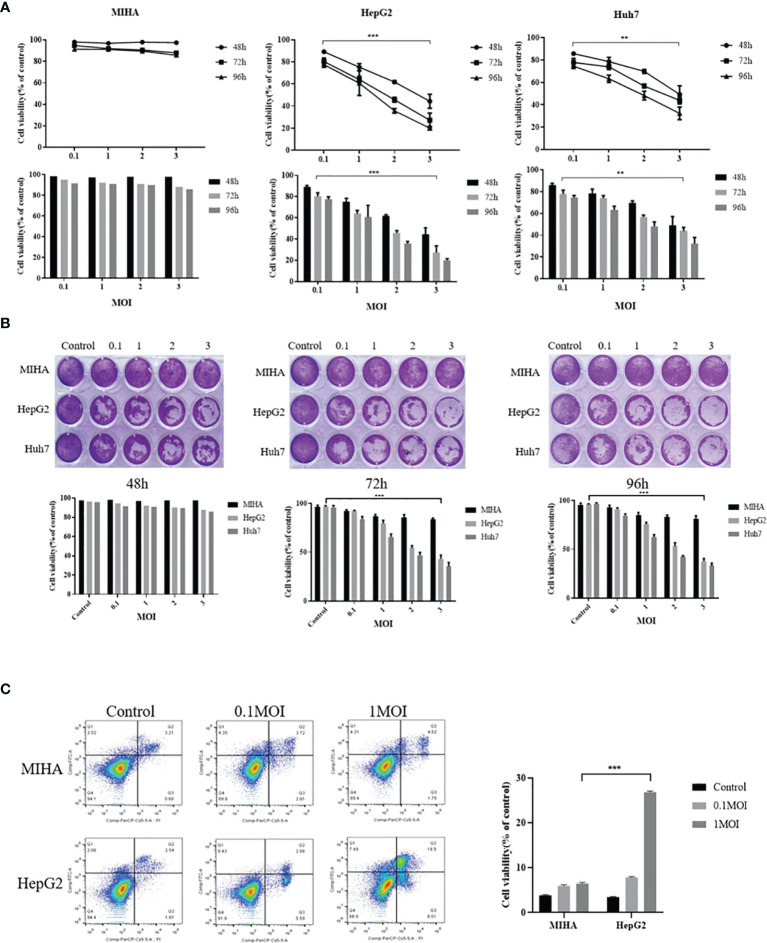
Selective cytotoxicity of rFlu-huCTLA4 on different hepatocellular carcinoma cell lines *in vitro*. **(A)** MTS showed that rFlu-huCTLA4 had no effect on MIHA but had a selective killing effect on HepG2 and Huh7. The activity of HepG2 cells at 1, 2, and 3 MOI was significantly lower than that at 0.1 MOI 72 h after infection; however, Huh7 cells only 2 and 3 MOI decreased significantly compared with 0.1 MOI cells. **(B)** The rFlu-huCTLA4 recombinant oncolytic virus detected by crystal violet can significantly inhibit the activity of hepatoma cells but has no effect on normal hepatocytes, which is consistent with the results of MTS. **(C)** Flow cytometry showed that recombinant oncolytic virus rFlu-huCTLA4 could induce apoptosis of HepG2 cells in a dose-dependent manner. At MOI of 1, the effect of apoptosis of HepG2 cells was more significant than that of MIHA. (**P<0.01,***P<0.001).

The rFu-huCTLA4 was used to infect MIHA, HepG2, and Huh7 cells at 0.1, 1, 2, and 3MOI. The cell viability was detected by MTS after 48, 72, and 96 h of infection. The results showed that the effect of rFlu-huCTLA4 on MIHA cells had no significant change, but the killing power of rFlu-huCTLA4 on HepG2 and Huh7 increased significantly with the time and dose increases. It was demonstrated that rFlu-huCTLA4 can selectively kill hepatocellular carcinoma cells in a time- and dose-dependent manner. It was notable that rFlu-huCTLA4 at 1 MOI caused a significant decrease in HepG2 cell activity than 0.1 MOI after 72 h of viral infection. However, no obvious difference was observed in Huh7 cells (**Figure 3A**).

To further clarify the oncolytic effect of the rFlu-huCTLA4 virus on hepatoma cell lines and normal liver cells, the crystal violet test was performed. Inconsistent with the MTS assay, crystal violet staining showed that the virus could effectively and specifically kill hepatoma cells without damaging normal liver cell lines, in a time- and dose-dependent manner (**Figure 3B**).

Next, we examined the cell apoptosis of rFlu-huCTLA4 on MIHA and HepG2 cells with flow cytometry. As the flow cytometry result shown in **Figure 3C**, the apoptosis rate of HepG2 cells at MOI 0.1 and 1 was 7.84 ± 0.17% and 26.76 ± 0.26%, respectively, and 3.45 ± 0.1% in the control group after 48 h postinfection.

### T-Cell Activations Induced by rFlu-huCTLA4 *In Vivo*

**Figure 4:**
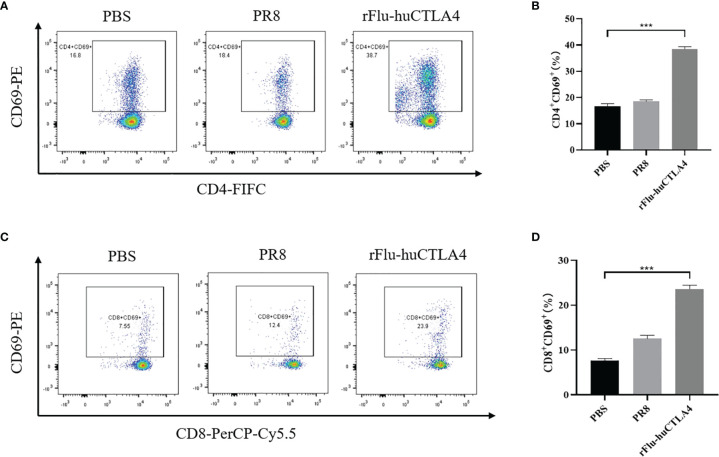
rFlu-huCTLA4 caused potential cancer immunotherapy in the H22 cell tumor-bearing model. Flow cytometric analysis of infiltrated CD4+CD69+ T cells **(A, B)** and CD8+ CD69+ T cells **(C, D)** in the spleen of the tumor-bearing BALB/c mice with rFlu-huCTLA4 treatment. The percentage of CD4+ CD69+ T cells and CD8+ CD69+ T cells in the spleen of tumor-carrying BALB/c mice treated with rFlu-huCTLA4 was higher than that of the PBS group and PR8 group. (***P<0.001).

BALB/c mice were used to construct a subcutaneous tumor-bearing model of mouse hepatocellular carcinoma H22 cells to detect the systemic antitumor immune response. The involvement of cytotoxic T cells (CTL, CD8^+^ T cells) and helper T cells (CD4^+^ T cells) is the most critical factor in tumor immunotherapy. CD8+ T cells can be targeted to kill cancer cells, while CD4^+^ T cells can maintain and enhance the underlying immune function. In addition, CD69^+^ is the earliest induced cell surface glycoprotein, which is the early activation state of T cells and participates in T-cell immune activities. Spleen is an important immunomodulatory organ in the body and contains a large number of lymphocytes, which can be used to detect T-cell activation status. Thus, T-cell activation in the spleen was further analyzed on day 7 after the last drug treatment. The percentage of CD8^+^ CD69^+^ T cells in the spleen of mice was examined and analyzed with flow cytometry. Results showed that the percentage of CD4^+^ CD69^+^ T cells in the spleen of rFlu-huCTLA4-treated mice increased significantly to 38.7%, which was higher than that in PBS- (16.8%) or PR8-treated (18.4%) mice. Meanwhile, the rFlu-huCTLA4 treatment effectively increased the percentage of CD4^+^ CD69^+^ T cells in the rFlu-huCTLA4 treatment group (**Figures 4A, B**). As shown in **Figures 4C, D**, the percentage of CD8^+^ CD69^+^ T cells in the spleen of rFlu-huCTLA4-treated mice increased significantly to 23.9%, which was higher than that in PBS- (7.55%) or PR8-treated (12.4%) mice. Neither PBS injection nor PR8 treatment promoted the activation of CD8^+^ CTL in the spleen. These results showed that T-cell infiltration in the spleen of mice was enhanced and activated after rFlu-huCTLA4 treatment.

### Pathological Changes of the Tumor-Bearing Model of Mice Treated With rFlu-huCTLA4

**Figure 5:**
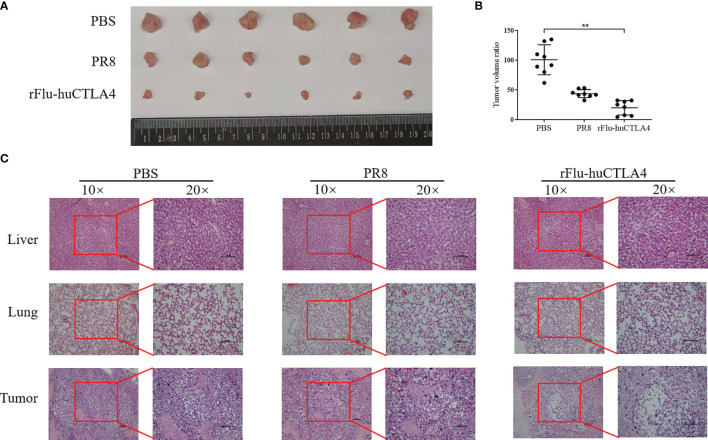
The tumor was isolated and the pathological changes observed. **(A, B)** After 7 days of inoculation, the tumor tissue of mice was isolated, and the tumor tissue of the rFlu-huCTLA4 group was significantly smaller than that of the PBS and PR8 group. **(C)** After being inoculated with rFlu-huCTLA4, obvious necrosis and dissolution were observed in tumor pathological sections of mice, and no abnormality was observed in liver and lung tissues. Also, there was no significant change in PBS and PR8 groups. (**p<0.01).

After 7 days of the last inoculation, the liver, lung, and tumor tissues of mice were isolated and the tumor tissue sizes of each group were compared. We found that the tumor sizes in the rFlu-huCTLA group were significantly smaller than those of the PBS and PR8 groups (**Figures 5A, B**). The liver, lung, and tumor tissues of the mice were stained with pathological HE staining, and the results are shown in **Figure 5C**. As expected, no viral load was detected in all tissues of mice in the PBS control group. Meanwhile, we performed HE staining of the tumor tissue of mice and found that tumor sites in the rFlu-huCTLA4 group exhibited significant necrosis, with pyknosis and fragmentation of nuclei and more chronic inflammatory cell infiltration into the interstitium. On the contrary, no obvious cancer cell necrosis was observed in the tumor site of mice in the PBS group and the PR8 group.

### Oncolytic Effect of rFlu-huCTLA4 Virus in the PDX Model

**Figure 6:**
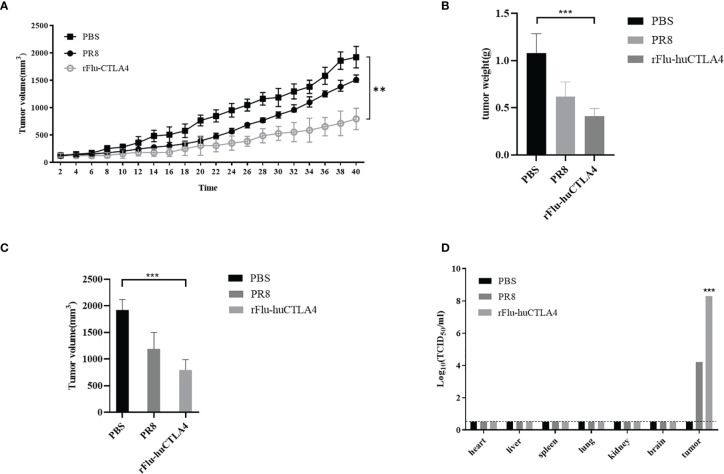
Oncolytic effect of recombinant virus rFlu-huCTLA4 *in vivo*. **(A)** PBS, PR8, and rFlu-huCTLA4 were injected respectively. The tumor volume was measured every 2 days, and the difference was significant (p < 0.01). rFlu-huCTLA4 could significantly inhibit the growth of the tumor. **(B)** 40 days later, the PDX mice were killed, tumors were isolated, and tumor volume was measured. rFlu-huCTLA4 significantly inhibited tumor volume growth. **(C)** Weighing the tumor, rFlu-huCTLA4 significantly inhibited tumor weight growth. **(D)** The heart, liver, spleen, lung, kidney, brain, and tumor tissue were isolated and viral load measured. (**P<0.01,***P<0.001).

As seen in **Figure 6A**, the tumor volume of PDX mice inoculated with PBS or rFlu-huCTLA4 was measured every 2 days. The tumor volume of mice in the rFlu-huCTLA4 group increased more slowly than that of the PBS group. After 40 days post administration, there was a significant difference in tumor volume of mice between the control group and the experimental group (p < 0.01). The tumor volume and weight of the rFlu-huCTLA4 group were significantly smaller than those of the control group (**Figures 6B, C**). The high viral load was detected in the tumor tissues of mice in the rFlu-huCTLA4 group, whereas no virus was found in the heart, liver, spleen, lung, kidney, or brain tissues (**Figure 6D**). The results showed that rFlu-huCTLA4 could significantly decrease the tumor volume and weight in the liver cancer PDX animal model as indicated by higher viral loads and obvious pathological alterations.

## Discussion

Although there are many clinical treatment strategies for liver cancer, the efficacy of these drugs is still far from satisfactory due to many reasons: (1) the occultness of liver cancer, the late stage when it is discovered; (2) individual differences in patients’ sensitivity to drugs; and (3) drug resistance of molecularly targeted drugs and other reasons. Researchers have focused on the immunotherapy of liver cancer. To enhance the sensitivity of the body to targeted drugs such as sorafenib, they have made various attempts and made some progresses, but there is still a gap from bench to clinical application (30, 31).

OVs are a novel oncologic agent with considerable flexibility that could induce tumor cell death while promoting congenital and tumor-specific adaptive immune responses (32). Up to now, four oncolytic virus products have been approved worldwide, and approximately 180 oncolytic virus projects are under development worldwide (https://ClinicalTrials.gov). CTLA4 plays a key role in regulating immune response and inducing self-tolerance. CTLA4 controls T-cell activation in several ways, which is important for protecting autoimmunity (33). This inhibitory molecule defends autoimmunity not only by inhibiting the activation and function of autoreactive T cells but also by inhibiting the differentiation of Th17 and promoting the proliferation of Treg cells (34). Therefore, the CTLA4 pathway regulates the balance between autoreactive T cells and Treg, and the decrease of CTLA4 expression or function may lead to the occurrence of autoimmune diseases (35, 36). The CTLA4 antibody can effectively block the expression of CTLA4, can effectively and specifically inhibit cellular and humoral immune responses *in vitro* and *in vivo*, with very low toxicity, and is considered a promising new immunosuppressive drug (37). In particular, ipilimumab approved by the FDA for clinical treatment in the United States has achieved good efficacy in the treatment of advanced metastatic melanoma and other tumors (38).

At present, the popular accepted explanation for the mechanism of oncolytic viruses is given below. The most of viewpoints are that oncolytic viruses proliferate massively in tumor cells through self-replication to lysis tumor cells and then release more oncolytic viruses to cause the tumor lysis. Then, a large number of virus-pathogen-related molecular patterns (PAMP) and cell-derived DAMPs are released, which attract and activate antigen-presenting cells (APC). The tumor-related antigen is activated, after entering the draining lymph nodes from the periphery, and differentiate into mature DC. The virus antigen was presented through MHC I and class II to activate CD4+ and CD8+ T cells which stimulate the body to produce T-cell responses to produce tumor-specific immune responses for tumor regression. Herein, the mechanism of action of the Flu-huCTLA4 virus selectively killing cancer cells was needed to be explored in our deep investigations, for example, the inclusion of additional mechanistic studies (immune cell depletions, etc.).

In this study, we firstly constructed the recombinant PB1 and PA plasmids of influenza virus and successfully rescued the chimeric oncolytic virus carrying the human CTLA4 antibody rFlu-huCTLA4 using reverse genetics. The HA titer of P1-generation rFlu-huCTLA4 was 2^7^~2^8^, and the viral titer was 10^8~9^ TCID_50_/ml. The chicken embryo passage experiment showed that the HA titer was stable at 2^8^~2^9^ from P1 to P5. The morphology of the rFlu-huCTLA4 virus was consistent with the influenza virus, and the size of the virus was distributed between 80 and 120 nm. ELISA assays showed that the huCTLA4 antibody level in each chicken embryo was 0.45 ± 0.03 g at 72 h post vaccination. The virus titers in HepG2 and MDCK cells began to increase at 12 h, reached the peak value of 2^7^ at 72 h, and decreased to 2^6^ at 96 h postinfection. Flow cytometry displayed that rFlu-huCTLA4 could induce HepG2 cell apoptosis in a dose-dependent manner, especially at the value of 1 MOI; HepG2 cell apoptosis rates were more significant than those of MIHA cells. The oncolytic virus, rFlu-huCTLA4, has been confirmed to have a clear role in targeting killing of hepatocellular carcinoma by HepG2 cells *in vitro*. We will explore the broad-spectrum killing effect in Huh7 cells and other types of tumors in our subsequent studies. H22 model mice bearing tumors shows that the rFlu-huCTLA4-treated group activated cytotoxic CD8^+^ T cells and CD4^+^ T cells in the spleen of mice, suggesting that rFlu-huCTLA4 can trigger the mechanism of immune system activation *in vivo*. In particular, activation of CD8^+^ T cells can directly kill targeted cancer cells, which may confer effective antitumor ability with rFlu-huCTLA4. Liver PDX mice showed that the rFlu-huCTLA4 virus could significantly inhibit tumor growth of mice *in vivo*. Of course, this study has several limitations. For example, the antitumor mechanisms of action of OVs remain to be fully elucidated. Whether the oncolytic virus rFlu-huCTLA4 has a broad-spectrum killing effect on other solid tumor cells remains to be further investigated in the following research. The rFlu-huCTLA4 virus with immune-checkpoint inhibitors in terms of therapeutic efficacy in *in vitro* and *in vivo* HCC models warrants further studies in the future. In conclusion, we have successfully generated the rFlu-huCTLA4 virus and confirmed its oncolytic efficacy to target hepatoma cells.

OVs will play a critical role in cancer treatment in the future and has a good promising potential in cancer vaccine development. However, there are still some problems faced with clinical application, such as the selection of the optimal virus for oncolytic virus therapy, as well as the optimal dose, route of administration, and regimen which need further research. Another challenge is to optimize the use of oncolytic viruses in combination with chemotherapy or immunotherapy drugs in oncology therapy to achieve a better clinical therapeutic effect.

## Data Availability Statement

All datasets generated for this study are included in the article. Further inquiries can be directed to the corresponding authors.

## Ethics Statement

The animal study was reviewed and approved by the Animal Ethics Committee of the Fifth Medical Center of the Chinese People’s Liberation Army General Hospital.

## Author Contributions

HY and GL performed the experiments and wrote the draft manuscript. FS, JC, and JY collected the data. HY, GL, SZ, and PY analyzed the data. All authors contributed to the article and approved the submitted version.

## Funding

This work was supported by the Beijing Natural Science Foundation from the Chinese government (Grant No. 7202194).

## Conflict of Interest

The authors declare that the research was conducted in the absence of any commercial or financial relationships that could be construed as a potential conflict of interest.

## Publisher’s Note

All claims expressed in this article are solely those of the authors and do not necessarily represent those of their affiliated organizations, or those of the publisher, the editors and the reviewers. Any product that may be evaluated in this article, or claim that may be made by its manufacturer, is not guaranteed or endorsed by the publisher.
